# Association of Social Determinants, Multimorbidity, and Functional Status with Mortality after Pneumonia

**DOI:** 10.1093/geroni/igab046.2381

**Published:** 2021-12-17

**Authors:** Chan Mi Park, Hye Chang Rhim, Eun Sik Lee, Wonsock Kim, Jong Hun Kim, Dae Kim

**Affiliations:** 1 Harvard T.H.Chan School of Public Health , Brookline, United States; 2 Harvard University, Orlando, Florida, United States; 3 Korea University Anam Hospital, Korea University Anam Hospital, Seoul-t'ukpyolsi, Republic of Korea; 4 Eulji Medical Center, Eulji Medical Center, Kyonggi-do, Republic of Korea; 5 CHA Bundang Medical Center, CHA Bundang Medical Center, Kyonggi-do, Republic of Korea; 6 Hebrew SeniorLife, Boston, Massachusetts, United States

## Abstract

Social support, multimorbidity, and functional status are important determinants of health in older adults, but their prognostic implications remain unclear after an acute illness. We conducted a prospective cohort study of 201 patients 65 years or older who were hospitalized for pneumonia at a university hospital in Korea in 2019-2020. K-means cluster analysis was performed using social deprivation score (range: 0-5), activities of daily living (range: 0-7), instrumental activities of daily living (range: 0-7), physical limitation score (range: 0-7), and Gagne comorbidity index (range: 0-24) (higher scores indicate higher risk). Four groups were identified: 1) Group A: physically limited and non-disabled group with limited social support; 2) Group B: multimorbid but functional group with social support; 3) Group C: multimorbid and disabled group with social support; 4) Group D: multimorbid and disabled group with limited social support. For Groups A through D, the Kaplan-Meir estimates for 6-month mortality were 10.0%, 18.0%, 34.2%, and 43.6%, respectively, and the 6-month mean survival times were 166.4 days (95% CI: 156.1-176.6), 156.9 days (95% CI: 140.8-173.1), 145.2 days (95% CI: 126.6-163.8), and 125.9 days (95% CI: 107.7-144.1), respectively. After adjusting for sex, age, and pneumonia severity score, the hazard ratios for Groups B through D versus Group A were 2.07 (95% CI: 0.70-6.13), 3.14 (95% CI: 1.17-8.42), and 4.38 (95% CI: 1.73-11.04), respectively. Our results suggest that multimorbidity and disabilities were implicated in higher risk of 6-month mortality after pneumonia, and social support may mitigate this risk among those with multimorbidity and disability.

